# Does self-reported symptom questionnaire play a role in nonadherence to colonoscopy for risk-increased population in the Tianjin colorectal cancer screening programme?

**DOI:** 10.1186/s12876-021-01701-z

**Published:** 2021-03-09

**Authors:** Lizhong Zhao, Xiaorui Zhang, Yongjie Chen, Yuan Wang, Weihua Zhang, Wenli Lu

**Affiliations:** 1 Department of Gastroenterology, Tianjin Union Medical Center, Tianjin, China; 2 Department of Epidemiology and Health Statistics, Tianjin Medical University, Tianjin, China

**Keywords:** Colonoscopy, Colorectal cancer, Screening, Early diagnosis

## Abstract

**Background:**

A colorectal cancer screening programme (CCSP) was implemented from 2012 to 2017 in Tianjin, China. Residents with a positive faecal immunochemical test (FIT) or positive self-reported symptom questionnaire (SRSQ) were recommended to undergo colonoscopy. The objective was to investigate the potential factors associated with nonadherence to colonoscopy among a risk-increased population.

**Methods:**

Data were obtained from the CCSP database, and 199,522 residents with positive FIT or positive SRSQ during two screening rounds (2012–2017) were included in the analysis. Logistic regression analysis was performed to assess the association between nonadherence to colonoscopy and potential predictors.

**Results:**

A total of 152,870 (76.6%) individuals did not undergo colonoscopy after positive FIT or positive SRSQ. Residents with positive SRSQ but without positive FIT were more likely not to undergo colonoscopy (negative FIT: OR, 2.35; 95% CI, 2.29–2.41, no FIT: OR, 1.27; 95% CI, 1.24–1.31). Patients without a cancer history were less likely to undergo colonoscopy even if they received risk-increased reports based on the SRSQ.

**Conclusion:**

In the CCSP, seventy-seven percent of the risk-increased population did not undergo colonoscopy. FIT should be recommended since positive FIT results are related to improved adherence to colonoscopy. Residents with negative FIT but positive SRSQ should be informed of the potential cancer risk to ensure adherence to colonoscopy.

## Background

Colorectal cancer (CRC) is the fourth most common cancer in China, and an estimated 180,000 Chinese patients died because of CRC in 2014 [1]. Routine screening can reduce the burden of this disease, and there are a number of screening modalities [2]. Faecal occult blood test/ faecal immunochemical test (FOBT/FIT) and risk assessments with questionnaires are recommended for CRC screening by the National Cancer Institute (NCI) [3]. However, the benefits of FOBT/FIT or risk assessment questionnaires cannot be achieved unless positive results are followed by colonoscopy [4]. Colonoscopy screening is widely considered the gold standard for CRC screening based on its ability to both visualize and remove polyps and neoplastic lesions in all regions of the colon [5]. Colonoscopy screening has been suggested to reduce CRC incidence rates and mortality rates [6–8], and it is recommended by relevant major organizational guidelines [9–11]. Despite the strong evidence of decreasing CRC incidence and mortality, low rates (23.1–50%) of colonoscopy follow-up are common in the colorectal screening [4, 12–14].

Colonoscopy screening is associated with a reduction in CRC incidence and mortality [15]. However, the benefits of colonoscopy cannot be achieved if the risk-increased population does not undergo colonoscopy. The CRC screening of Alberta’s Tomorrow Project showed that colonoscopy adherence was associated with screening patterns [16]. The colorectal symptoms and symptom combination also contribute to colonoscopy adherence [17]. A colorectal cancer screening programme (CCSP) was carried out from 2012 to 2017 in Tianjin, China. The aim of this analysis was to assess adherence to follow-up colonoscopy after positive FIT or positive SRSQ and potential predictors associated with nonadherence.

## Methods

### Colorectal cancer screening programme in Tianjin, China

**Fig. 1:**
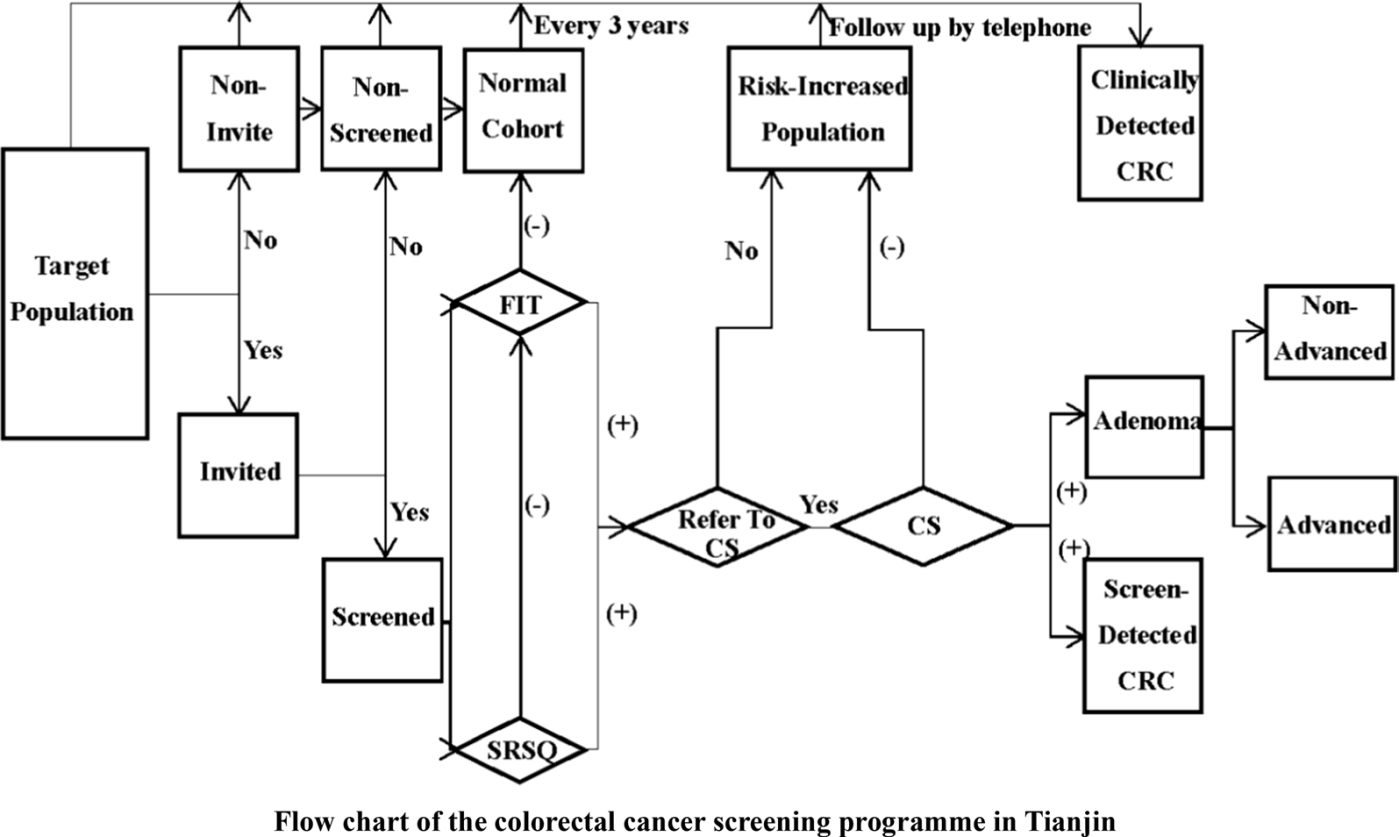
Flow chart of the colorectal cancer screening programme in Tianjin

The CCSP was conducted in Tianjin, a Chinese city with a population of 15.57 million [18]. According to the *Technical Plan for Early Diagnosis and Early Treatment of Colorectal Cancer* formulated by the National Health Commission of the People’s Republic of China, a colorectal cancer screening programme was conducted by the Tianjin CRC screening group in 2012. The first round was from 2012 to 2014, and the second round was from 2015 to 2017. Individuals aged 40–74 years were invited to complete a questionnaire and FIT in a local screening programme centre. Then, colonoscopy was recommended for residents with positive FIT or positive SRSQ. Individuals who did not undergo colonoscopy on the appointment day were followed up by telephone in the next year (Fig. 1).

### Study population

All participants targeted by the programme with positive FIT or positive SRSQ results during either of the two screening rounds (2012–2014 or 2015–2017) were eligible for inclusion in the present study.

### Positive SRSQ and subgroups

The structured questionnaire included questions on nine topics: chronic diarrhoea history; chronic constipation history; mucus or blood stool history; chronic appendicitis or appendectomy; chronic gallbladder disease or gallbladder surgery history; stressful life event over the past 2 decades; cancer history; colon polyp history; or family history of CRC among first-degree relatives. Subjects who had any first-degree relatives with CRC cancer, who had ever been affected by polyps or cancer or who had ≥ 2 of the following clinical syndromes, chronic constipation, chronic diarrhoea, bloody mucus, history of negative life events, history of chronic appendicitis or appendectomy, history of chronic gallbladder disease or gallbladder surgery, were defined as positive on the SRSQ.

**Fig. 2:**
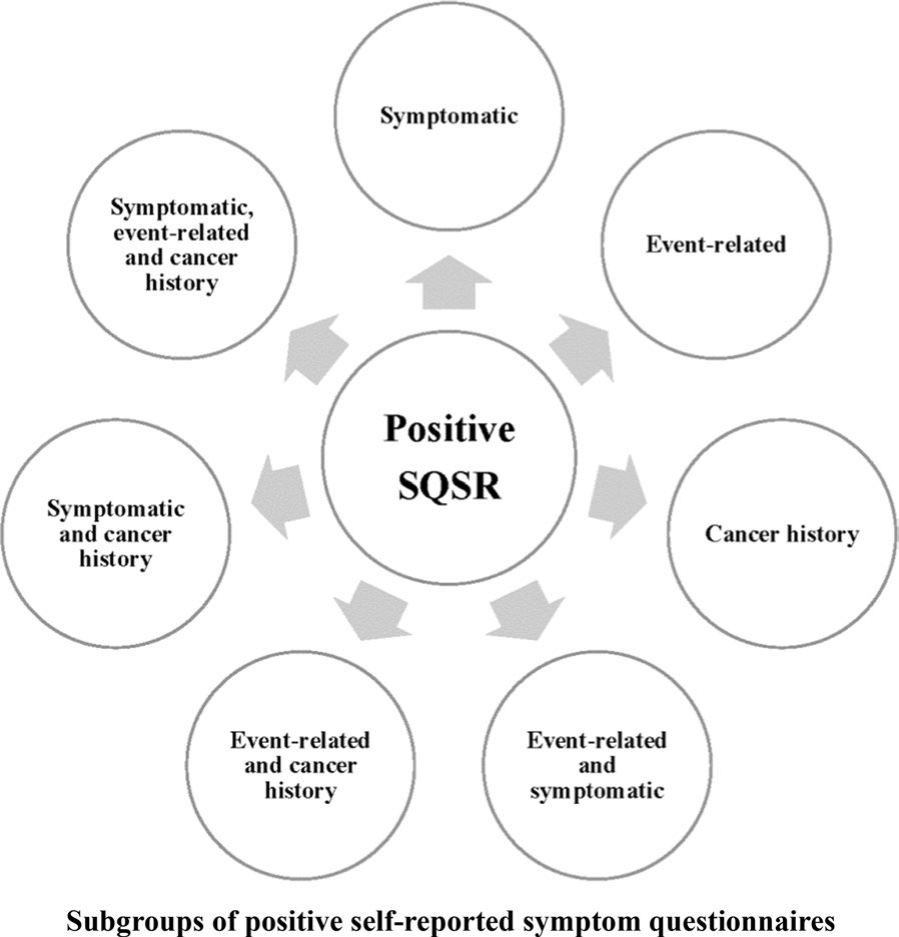
Subgroups of positive self-reported symptom questionnaires

Then, we classified the risk-increased participants who had a positive questionnaire response into 7 subgroups based on the nine questions (Fig. 2). (1) Symptomatic participants based on chronic constipation, chronic diarrhoea, and bloody mucus; (2) Event-related participants who reported ≥ 2 of the following: history of negative life events, history of chronic appendicitis or appendectomy, history of chronic gallbladder disease or gallbladder surgery; (3) Participants with cancer history who had any first-degree relatives with CRC cancer and who had ever been affected by polyps or cancer; (4) Event-related and symptomatic participants who had combined characteristics of (1) and (2); (5) Event-related and cancer history participants who had combined characteristics of (2) and (3); (6) Symptomatic and cancer history participants who had combined characteristics of (1) and (3); and (7) Symptomatic, event-related and cancer history participants who had combined characteristics of (1), (2) and (3).

### Positive faecal immunochemical test (FIT) and subgroups

Faecal samples were obtained from 4,215,405 subjects at their home using the collection kit provided by the manufacturer (ABON, China). Participants were asked to collect their stool and send it to the community hospital. No specific dietary restriction was stipulated. All tests were processed at the laboratory after collection. According to the manufacturer’s instructions, this qualitative test is considered positive when the sample is positive for haemoglobin.

Individuals were classified according to the FIT and SRSQ results, namely, positive SRSQ and positive FIT, positive SRSQ but negative FIT, positive FIT but negative SRSQ and positive SRSQ but negative FIT.

### Statistical analysis

Analyses were performed using SAS statistical software (version 9.4, SAS Institute, INC., Cary, North Carolina, USA). Categorical variables are described as numbers and percentages. Individuals were classified into two categories: colonoscopy and nonadherence to colonoscopy. Nonadherence to colonoscopy was defined as absence of records of colonoscopy after a positive SRSQ or a positive FIT through Dec 2018. Logistic regression analysis was used to assess the association between colonoscopy adherence and potential factors. The estimated associations are presented as odds ratios (*ORs*) and 95% confidence intervals (*CIs*). All estimations were adjusted for age, sex, education level and marital status. The criterion for statistical significance was set as *ɑ* ≤ 0.05 (2 size tailed).

## Results

### Study population

**Fig. 3:**
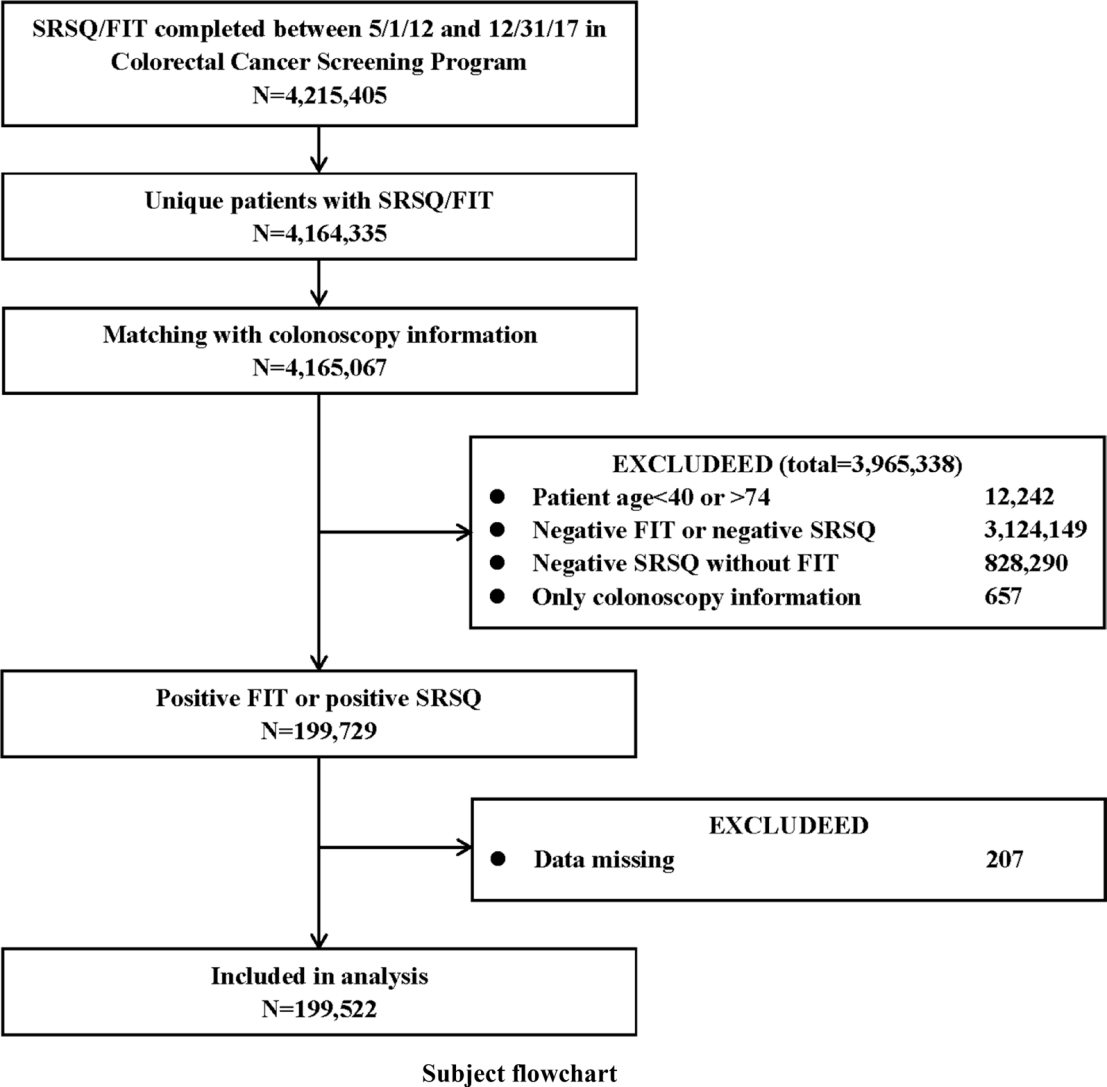
Subject flowchart

There were 4,215,405 CCSP participants from 2012 to 2017. Among the 199,729 participants with positive SRSQ or positive FIT, 207 were excluded because of missing data, and 199,522 were analysed (Fig. 3). A total of 152,870 (76.6%) individuals did not undergo colonoscopy after positive FIT or positive SRSQ.

Table 1 reports the main study population characteristics. Most participants were sixty years old or above (57.90%), lived in rural settings (61.82%), had a partner (92.10%) and participated in the first round (61.32%). Women reported lower levels of education than men (63.13% vs 36.87%). Forty-one percent of participants had negative FIT but positive SRSQ.

**Table 1:** Characteristics of the risk-increased population in the CCSP 2012–2017

Characteristics	Men		Women		Total	
	n	%	n	%	N	%
Age						
40–49 years old	12,269	45.47	14,714	54.53	26,983	13.52
50–59 years old	23,701	41.57	33,318	58.43	57,019	28.58
60–74 years old	50,095	43.36	65,425	56.64	115,520	57.90
Education						
Elementary School/below	21,258	36.87	36,394	63.13	57,652	28.90
Secondary/middle School	54,455	44.86	66,936	55.14	121,391	60.84
College/above	10,352	50.55	10,127	49.45	20,479	10.26
Marital status						
Divorced	620	34.97	1153	65.03	1773	0.89
Widowed	2492	20.50	9665	79.50	12,157	6.09
Unmarried	952	52.11	875	47.89	1827	0.92
Married	82,001	44.62	101,764	55.38	183,765	92.10
Residential area						
Urban	31,586	41.47	44,584	58.53	76,170	38.18
Rural	54,479	44.17	68,873	55.83	123,352	61.82
Round						
The first round	51,891	42.41	70,463	57.59	122,354	61.32
The second round	34,174	44.29	42,994	55.71	77,168	38.68
Risk-increased population						
SRSQ+ and no FIT	21,345	42.20	29,241	57.80	50,586	25.35
FIT− and SRSQ+	32,867	39.84	49,634	60.16	82,501	41.35
FIT+ and SRSQ−	27,071	48.44	28,809	51.56	55,880	28.01
FIT+ and SRSQ+	4782	45.31	5773	54.69	10,555	5.29

The results showed that young participants were likely not adhering to colonoscopy (40–49 years old (78.50%)). Shorter education duration was associated with not adhering to colonoscopy after positive primary screening (elementary school/below (79.88%), secondary/middle school (75.86%)). Compared with married participants, the others were significantly more likely to not adhere to colonoscopy (divorced (79.36%), widowed (84.21%), unmarried (79.09%)). Not adhering to colonoscopy was observed in participants reporting a positive questionnaire result but negative FIT or no FIT (positive questionnaire negative FIT (83.94%) and positive questionnaire with no FIT (75.32%)) (Table 2).

**Table 2:** Factors associated with nonadherence to colonoscopy in the risk-increased population in the CCSP 2012–2017

Characteristics	Nonadherence to CS		Univariate		Multivariate	
	*N*	*%*	*OR *(95.0% CI)	*P* value	*OR *(95.0% CI)	*P* value
Age						
60–74 years old	89,751	77.69	1.00		1.00	
40–49 years old	21,182	78.50	1.05 (1.02–1.08)	0.004	1.19 (1.15–1.23)	< 0.001
50–59 years old	41,937	73.55	0.80 (0.78–0.82)	< 0.001	0.81 (0.79–0.83)	< 0.001
Education						
College/above	14,728	71.92	1.00		1.00	
Elementary School/below	46,051	79.88	1.55 (1.49–1.61)	< 0.001	1.81 (1.73–1.88)	< 0.001
Secondary/middle School	92,091	75.86	1.23 (1.19–1.27)	< 0.001	1.35 (1.30–1.39)	< 0.001
Sex						
Female	88,616	78.11	1.00		1.00	
Male	64,254	74.66	0.83 (0.81–0.84)	< 0.001	0.91 (0.89–0.93)	< 0.001
Marital status						
Married	139,781	76.07	1.00		1.00	
Divorced	1,407	79.36	1.21 (1.08–1.36)	0.001	1.13 (1.00–1.27)	0.049
Widowed	10,237	84.21	1.68 (1.60–1.76)	< 0.001	1.44 (1.37–1.52)	< 0.001
Unmarried	1,445	79.09	1.19 (1.06–1.33)	0.003	1.16 (1.03–1.30)	0.012
Residential area						
Rural	94,416	76.54	1.00		1.00	
Urban	58,454	76.74	1.01 (0.99–1.03)	0.307	1.13 (1.10–1.15)	< 0.001
Round						
Second round	58,626	75.97	1.00		1.00	
First round	94,244	77.03	1.06 (1.04–1.08)	< 0.001	1.06 (1.04–1.09)	< 0.001
Risk-increased population						
FIT+ and SRSQ−	38,938	69.68	1.00		1.00	
SRSQ+ and no FIT	38,101	75.32	1.33 (1.29–1.36)	< 0.001	1.27 (1.24–1.31)	< 0.001
SRSQ+ and FIT−	69,250	83.94	2.27 (2.22–2.33)	< 0.001	2.35 (2.29–2.41)	< 0.001
SRSQ+ and FIT+	6,581	62.35	0.72 (0.69–0.75)	< 0.001	0.73 (0.70–0.76)	< 0.001

### Comparison of colonoscopy adherence according to SRSQ items

**Fig. 4:**
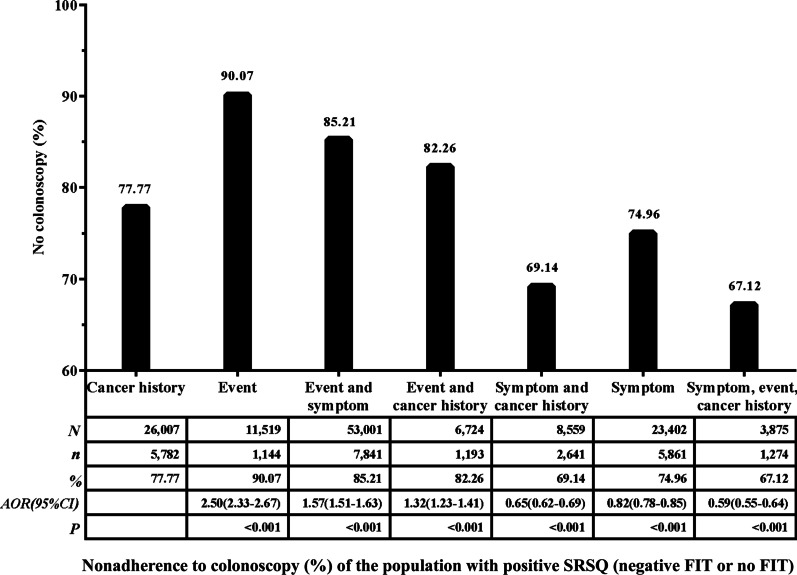
Nonadherence to colonoscopy (%) of the population with positive SRSQ (negative FIT or no FIT). Adjusted for age, sex, education level and marital status

**Fig. 5:**
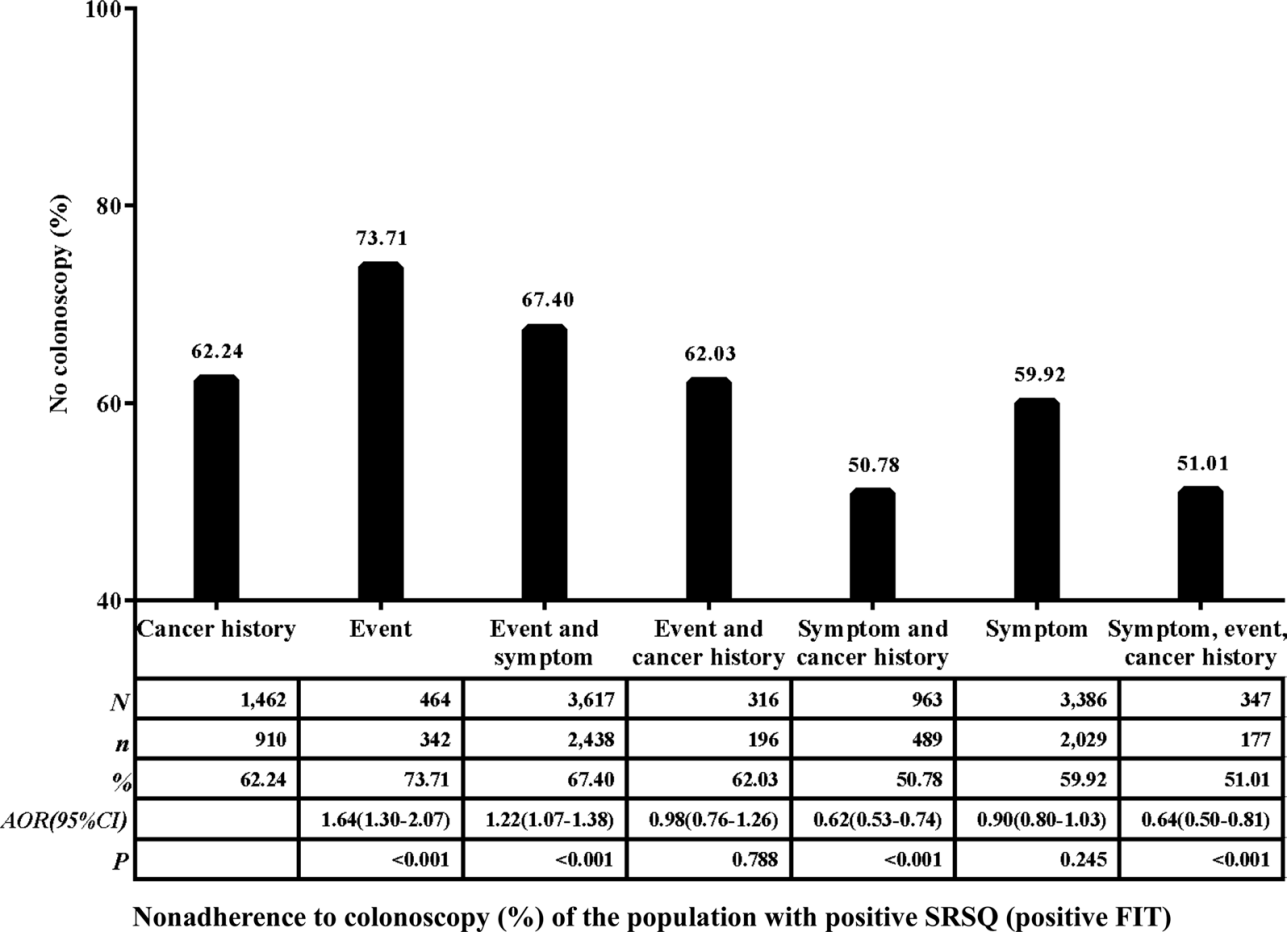
Nonadherence to colonoscopy (%) of the population with positive SRSQ (positive FIT). Adjusted for age, sex, education level and marital status

Solely event-related participants (who reported ≥ 2 of the following: history of negative life events, history of chronic appendicitis or appendectomy, history of chronic gallbladder disease or gallbladder surgery) had the highest nonadherence to colonoscopy (90.07%). The existence of symptoms promoted adherence to colonoscopy (Figs. 4, 5).

## Discussion

### Nonadherence to colonoscopy was high in the risk-increased population

Nonadherence to colonoscopy was high (76.6%) among 199,522 risk-increased participants in the CCSP between May 2012 and December 2017. The results were consistent with the results of a study conducted in Australia (70%) [19]. However, some studies contrast with our results. The rates of nonadherence to colonoscopy in the United States (38.3%) [20] and the United Kingdom (48.0%) [21] were lower than that in our study. In short, nonadherence to colonoscopy in China is still at a relatively high level.

### Demographic factors were associated with nonadherence to colonoscopy

Women and participants who were divorced, widowed, unmarried, had lower levels of education, participated in the first round, lived in an urban area and had a positive SRSQ result (negative FIT or no FIT) were associated with nonadherence to colonoscopy. The associations of colonoscopy adherence with age, sex, round, residential area, marital status and education have been studied before [13, 22–28]. Our finding that age [13, 27], marital status [23, 24, 29], education [13] and sex [28, 30–32] were associated with colonoscopy screening adherence is consistent with prior studies.

However, some of our findings contrast with findings from prior studies. From the perspective of the residential area, the urban population was more likely to not adhere to colonoscopy than the rural population, which was observed in our study and in others [33]. This situation may be related to the fact that rural residents are more likely to be organized. However, one prior study found that persons who lived in low-income urban areas were more likely to not adhere to colonoscopy than those who lived in high-income urban areas, and there were no statistically significant differences between rural and urban areas [34]. The differences in predictors of nonadherence to colonoscopy identified in our study from those in prior studies may be due to differences in the populations and settings studied.

In the first round, individuals aged 40–74 years who had positive FIT or SRSQ were invited to undergo colonoscopy. However, it is known from international data that colonoscopy does have a small but significant miss rate for polyps and even cancers [35]. Therefore, in the second round, only participants who had undergone a colonoscopy since their participation in the first round were excluded. This was done to assess the safety of the current exclusion practices and to determine the optimum protocol to maximize polyp and cancer detection [36]. After analysing our results from the second round and comparing them with the results from the first round of screening, we showed that, importantly, nonadherence to colonoscopy in the first round was higher than that in the second round. This result was also in line with other studies [28, 30, 37]. In contrast, a recent study showed the same adherence between round one and round two [36]. There was some increase between the two rounds, probably because the subjects who participated in the two rounds of screening did not repeat colonoscopy and the promotion was enhanced.

### Nonadherence to colonoscopy was related to positive SRSQ

We found that different risk-increased classifications of positive results in primary screening were associated with different adherence rates. When the primary screening result was positive SRSQ and negative FIT, nonadherence to colonoscopy was highest, which was similar to the results of a prior study in China [38]. Different risk-increased classification methods have been used in prior studies; for instance, a recent study by *Nathan M. Solbak* et al. showed risk classification based on personal CRC risk [16], in which nonadherence to colonoscopy was highest in average-risk participants. This situation may be related to the subject’s trust in the screening test. People are more convinced of the FIT results, and there is doubt about the results of the questionnaire; therefore, the risk-increased individuals who had negative FIT results were more likely to not adhere to colonoscopy in the CCSP.

Since nonadherence to colonoscopy was high when the primary screening result was positive SRSQ (negative FIT or no FIT), we performed subgroup analyses on the positive SRSQ (negative FIT or no FIT) and positive SRSQ with FIT groups. In this work, we show that the results of positive SRSQ (negative FIT or no FIT) and positive SRSQ with FIT groups are similar. The majority of patients who fell into the event-related population were more likely to not adhere to colonoscopy. This is important for colonoscopy screening, since knowledge of these issues (or lack of knowledge) will impact participants’ perceptions of their risk of getting cancer and their perceptions of the effectiveness or utility of undertaking the screening test [39]. Lack of knowledge about event-related risk factors might account for nonadherence to colonoscopy. Furthermore, our data revealed that nonadherence to colonoscopy significantly increased by adding symptomatic or cancer-related situations to event-related situations compared to symptomatic or cancer-related situations alone. The reason for this may be that in the combination of event-related situations and the other two situations, the event-related situation played a major role in reducing colonoscopy adherence. Therefore, the risk-increased population who had positive SRSQ based on event-related situations should be focused on in interventions.

Based on this study, we propose the following suggestions for improving colonoscopy adherence. First, from an individual perspective, we should improve the individual's understanding of screening tests and strengthen publicity and education. Second, from the perspective of hospital organization, the notification of the colonoscopy recommendation for the risk-increased population in the programme was performed by telephone. In a previous study [25], it was mentioned that the inconsistent expectation between doctors and patients was the main reason for reducing compliance, and the key to inconsistent expectations was poor communication between doctors and patients. There are also studies [25, 40] indicating that the communication of further screening between doctors and patients can improve colonoscopy adherence. Therefore, in subsequent practice, we can try to inform the subject to perform colonoscopy face to face, which may be an effective way to improve adherence to colonoscopy screening.

### Strengths and limitations of the study

This analysis was based on a large sample of patients and facilities. To our knowledge, this is the first analysis to explore the relationship between SRSQ and colonoscopy adherence. However, we were not able to assess physician, organization or environmental factors due to a lack of colonoscopy screening. Understanding the contribution of different perspectives and experiences will likely be critical to developing effective interventions to improve colonoscopy adherence. Additionally, the population in Tianjin is unique, and the findings may not generalize to other cities in China.

## Conclusion

We assessed the association between colonoscopy adherence and the primary screening method. FIT should be recommended since positive FIT results indicate increased risk and promote better adherence to colonoscopy. In addition, FIT alone may not be enough in CRC screening because FIT inevitably misses some important lesions that do not bleed or bleed intermittently. Individuals with a negative FIT but with a positive SRSQ must be informed of the potential risk of cancer to ensure adherence to colonoscopy. Finally, we performed further analysis and found that screening adherence was low when the risk-increased population was judged by event-related situations. Therefore, the risk-increased population who had positive SRSQ based on event-related situations should be focused on in interventions.

## Data Availability

The datasets used and/or analysed during the current study are available from the corresponding author on reasonable request.

## Abbreviations

CCSP: Colorectal cancer screening programme
FIT: Faecal immunochemical test
FOBT: Faecal occult blood test
SRSQ: Self-reported symptom questionnaire
CRC: Colorectal cancer
